# Neuropsychiatric Effects of Glucagon-Like Peptide-1 Receptor Agonists in Schizophrenia-Spectrum Disorders: A Systematic Review

**DOI:** 10.7759/cureus.114350

**Published:** 2026-08-11

**Authors:** Zakariya Y Alsanosi, Abdullah S Alshahrani, Abdulaziz A Nammazi, Omar A Alsunusi, Abdulrahman A Moafa, Ibtihal H Hadi, Ahmed A Alabdali, Abdullah W Alhaqbani, Razan A Shahat, Shahd A Albeladi, Aljoudi A Almajdoa

**Affiliations:** 1 Department of Psychiatry, Eradah Hospital, Jazan, SAU; 2 College of Medicine, King Khalid University, Abha, SAU; 3 College of Medicine, Jazan University, Jazan, SAU; 4 College of Medicine, Prince Sattam bin Abdulaziz University, Al-Kharj, SAU; 5 Clinical Pharmacy Program, College of Health Sciences, Al-Rayan Colleges, Madinah, SAU; 6 Faculty of Medicine and Surgery, Batterjee Medical College, Jeddah, SAU

**Keywords:** antipsychotics, cardiometabolic health, cognition, glp-1ras, glucagon-like peptide-1 receptor agonists, psychiatric symptoms, quality of life, randomised controlled trials, schizophrenia-spectrum disorders, systematic review

## Abstract

People with schizophrenia-spectrum disorders die 15-20 years prematurely, predominantly from cardiovascular disease, and antipsychotic-induced weight gain is a major, partly iatrogenic contributor to this excess mortality. Glucagon-like peptide-1 receptor agonists (GLP-1RAs) are potent weight-lowering agents that are increasingly used as metabolic adjuncts in this population, and preclinical evidence has suggested that they may also exert central, potentially pro-cognitive effects. However, their effects on psychiatric symptoms, cognition, and quality of life have not previously been systematically synthesized. We therefore conducted a systematic review following the Preferred Reporting Items for Systematic Reviews and Meta-Analyses (PRISMA) 2020 statement. Four databases (MEDLINE/PubMed, Cochrane Central Register of Controlled Trials (CENTRAL), Scopus, and Web of Science) were searched on 20 July 2026 for randomized controlled trials (RCTs) comparing any GLP-1RA with placebo, usual care, or an active comparator, added to antipsychotic treatment, in adults with schizophrenia-spectrum disorders. The primary outcome was psychiatric symptom severity, while secondary outcomes were cognition and quality of life or functioning. Reports were de-duplicated at the trial level using trial registration records, and risk of bias was assessed for each outcome domain using the revised Cochrane Risk of Bias tool for randomized trials (RoB 2). Because outcome measures were heterogeneous and variance data were largely unavailable, meta-analysis was not feasible, and findings were synthesized narratively according to the direction of effect. Eight reports representing four RCTs evaluating exenatide, liraglutide, and semaglutide (333 participants randomized) were included. No trial demonstrated a statistically significant effect on psychiatric symptom severity as measured by the Positive and Negative Syndrome Scale (PANSS), the Clinical Global Impression-Severity (CGI-S) scale, or the six-item Positive and Negative Syndrome Scale (PANSS-6). Likewise, neither of the two trials that assessed cognition, including one in which cognitive performance was the primary outcome, demonstrated a pro-cognitive effect. Quality-of-life outcomes were also largely unchanged, except for improved physical quality of life in the largest trial (36-Item Short Form Health Survey version 2 (SF-36v2) Physical Component Summary: +3.75, 95% confidence interval (CI) 1.52 to 5.98; P = .001), which a companion mediation analysis indicated was largely attributable to weight loss. Mental quality of life and functioning showed no significant improvements. Most outcome-domain assessments were judged to have some concerns regarding risk of bias, with none rated as high risk, and the certainty of the evidence was low to moderate. Overall, in adults with schizophrenia-spectrum disorders receiving antipsychotic treatment, GLP-1RAs showed no evidence of benefit or harm for psychiatric symptoms or cognition. Improvements in quality of life were limited to physical health and appeared to be mediated by weight loss rather than direct psychotropic effects. These findings support the role of GLP-1RAs as cardiometabolic therapies in this population, with no signal of psychiatric destabilization. Larger, adequately powered RCTs with neuropsychiatric primary outcomes, longer follow-up, harmonized outcome measures, and complete reporting of variance data are needed.

## Introduction and background

Schizophrenia-spectrum disorders are associated with substantial disability and markedly reduced life expectancy. Individuals with schizophrenia die 15-20 years earlier than the general population, with cardiovascular disease representing the leading cause of premature mortality [1-3]. This excess risk is driven by a high prevalence of obesity, dyslipidaemia, hypertension, and dysglycaemia, which are further compounded by the metabolic adverse effects of antipsychotic medications [2,4]. Although antipsychotics remain the cornerstone of treatment, agents such as clozapine and olanzapine are particularly associated with weight gain and metabolic dysfunction, creating a major challenge in balancing psychiatric benefits against long-term cardiometabolic risks [3-6].

Current strategies for managing antipsychotic-induced weight gain provide only modest benefits. Lifestyle interventions are often difficult to sustain, and metformin, the most established pharmacological option, produces relatively limited weight reduction. Consequently, there is growing interest in more effective metabolic therapies [1,4].

Glucagon-like peptide-1 receptor agonists (GLP-1RAs) have transformed the treatment of obesity and type 2 diabetes mellitus through their potent effects on weight reduction and cardiometabolic health [1,5]. In individuals receiving antipsychotic treatment, GLP-1RAs have also been shown to reduce body weight and improve metabolic outcomes, supporting their use as adjunctive therapy for antipsychotic-induced weight gain [2,4].

Beyond their metabolic effects, GLP-1RAs may influence brain function. Glucagon-like peptide-1 receptors are widely expressed throughout the central nervous system (CNS), and preclinical studies have demonstrated neuroprotective, anti-inflammatory, and synaptic effects [2,4,5]. These findings have prompted investigation of GLP-1RAs in neurological disorders and raise the possibility that they may also influence psychiatric symptoms, cognition, or quality of life in schizophrenia-spectrum disorders. However, evidence regarding these neuropsychiatric effects remains uncertain [3,5,6].

Although several randomized controlled trials (RCTs) have evaluated GLP-1RAs in adults with schizophrenia-spectrum disorders receiving antipsychotic treatment, neuropsychiatric outcomes have been reported only as secondary or exploratory endpoints, and their findings have not been systematically synthesized. Therefore, we conducted a systematic review evaluating the effects of GLP-1RAs on psychiatric symptoms, cognition, and quality of life in adults with schizophrenia-spectrum disorders receiving antipsychotic treatment.

## Review

Methods

Study Design

This systematic review was conducted and reported in accordance with the Preferred Reporting Items for Systematic Reviews and Meta-Analyses (PRISMA) 2020 statement [7]. The review question was structured using the Population, Intervention, Comparator, and Outcome (PICO) framework [8].

Eligible studies were RCTs, including prespecified secondary and post hoc analyses, evaluating GLP-1RAs added to antipsychotic treatment in adults (≥18 years) with schizophrenia-spectrum disorders. Comparators included placebo, usual care, or an active comparator. The primary outcome was psychiatric symptom severity, while secondary outcomes included cognition and quality of life or functioning.

Search Strategy and Study Selection

MEDLINE (via PubMed), the Cochrane Central Register of Controlled Trials (CENTRAL), Scopus, and Web of Science Core Collection were searched on 20 July 2026. Search terms combined GLP-1RAs, schizophrenia-spectrum disorders, and antipsychotic treatment. The search strategy combined terms related to GLP-1 receptor agonists (including exenatide, liraglutide, and semaglutide), schizophrenia-spectrum disorders, and antipsychotic treatment, with database-specific adaptations applied where appropriate (Appendices).

Records were screened by title and abstract, followed by full-text assessment against predefined eligibility criteria. To avoid double-counting participants, multiple publications arising from the same trial were identified using trial registration numbers and treated as a single study. Protocol papers were excluded from the synthesis but were used to verify prespecified methods and outcomes. Studies including mixed diagnostic populations were excluded unless data for participants with schizophrenia-spectrum disorders were reported separately. Reasons for full-text exclusion were recorded.

Data Extraction

Data were extracted using a standardized, piloted form. Extracted information included study design, participant characteristics, interventions, comparators, treatment duration, sample size, and funding sources. Outcome data included measurement instruments, sample sizes, effect estimates, 95% confidence intervals (CIs), P values, and analysis populations. Missing data were recorded as not reported and were not imputed.

Risk-of-Bias Assessment

Risk of bias was assessed for each outcome domain using the revised Cochrane Risk of Bias tool for randomized trials (RoB 2) [9]. Assessments covered the randomization process, deviations from intended interventions, missing outcome data, outcome measurement, selection of the reported result, and overall risk of bias. Because outcome measurement differed across psychiatric symptoms, cognition, and quality-of-life outcomes, each domain was assessed separately.

Data Synthesis

The feasibility of meta-analysis was evaluated by considering the number of available studies, comparability of outcome measures, availability of variance data, and clinical and methodological heterogeneity. Meta-analysis was not performed because too few studies reported comparable outcomes with sufficient statistical data for pooling, and substantial heterogeneity existed across interventions, outcome measures, and study designs.

Studies were grouped by outcome domain, and results were summarized according to the direction and statistical significance of effects, with reported effect estimates and 95% CIs presented where available. The certainty of evidence was assessed narratively using the Grading of Recommendations Assessment, Development and Evaluation (GRADE) framework [10].

Results

Study Selection

**Figure 1:**
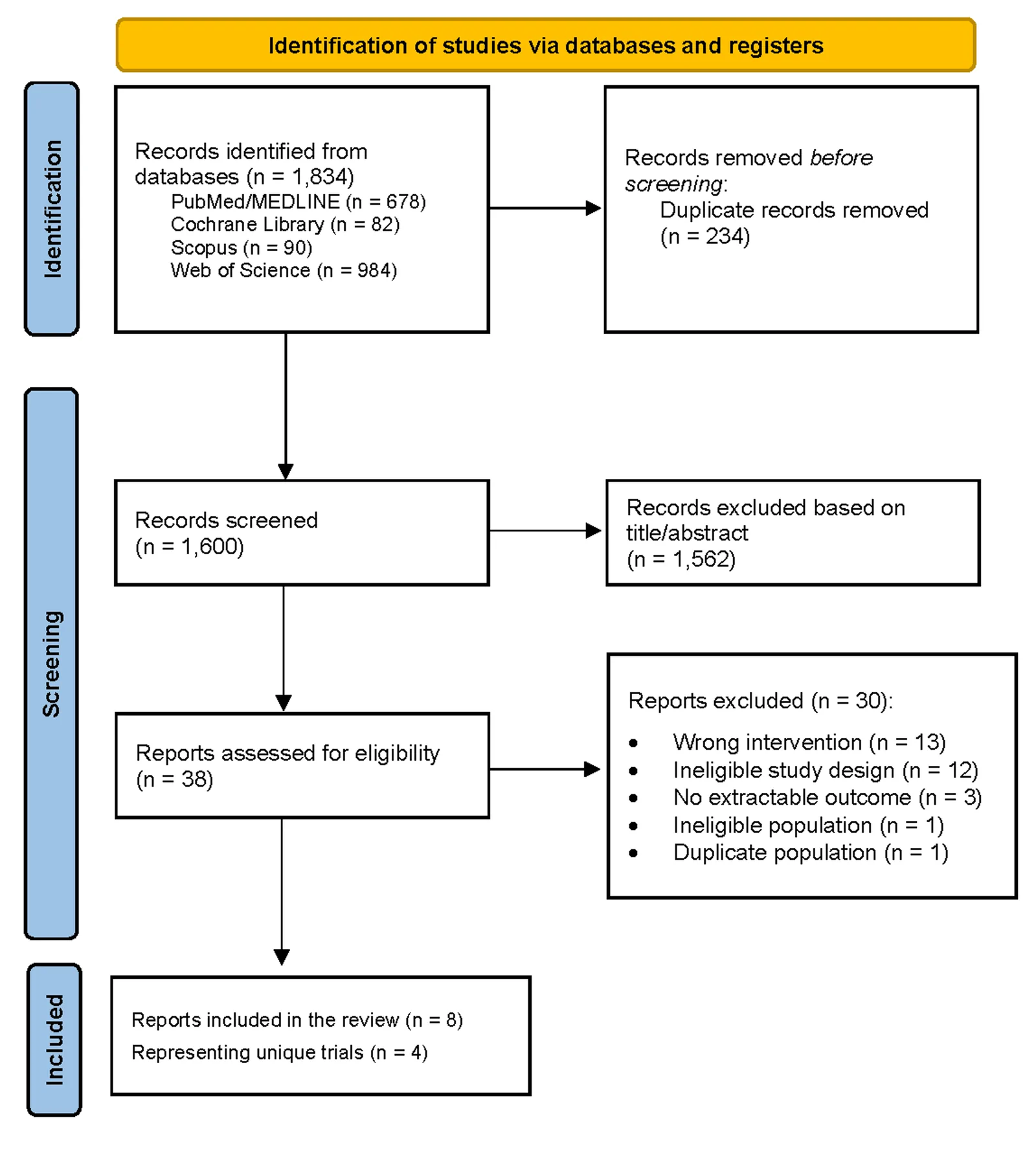
PRISMA flow diagram depicting the study selection process for the systematic review Following the identification of records through database searching and other sources, duplicates were removed, and the remaining records were screened. Full-text articles were assessed for eligibility, with exclusions documented along with reasons. Studies meeting the inclusion criteria were included in the final synthesis. PRISMA: Preferred Reporting Items for Systematic Reviews and Meta-Analyses

The database searches conducted on 20 July 2026 identified 1,834 records (MEDLINE/PubMed, n=82; Cochrane Central Register of Controlled Trials (CENTRAL), n=90; Scopus, n=678; Web of Science, n=984). After removal of 234 duplicates, 1,600 records underwent title and abstract screening, and 38 reports were assessed at full text. Thirty reports were excluded because of an ineligible intervention (n=13), ineligible population (n=12), ineligible outcome (n=3), mixed diagnostic populations without a separate schizophrenia-spectrum subgroup (n=1), or absence of outcome data (n=1). Eight reports representing four unique randomized controlled trials (RCTs) were included: COaST, TAO, the Larsen liraglutide trial, and HISTORI. Multiple publications arising from the same trial were considered linked reports and were grouped under their parent trial to avoid double-counting participants. Therefore, the unit of synthesis was the unique RCT rather than the individual publication. The included reports comprised primary trial publications and prespecified secondary or post hoc analyses derived from the same participant cohorts (Figure 1; Table 1).

**Table 1:** Characteristics of randomized controlled trials evaluating glucagon-like peptide-1 receptor agonists in patients with schizophrenia-spectrum disorders treated with antipsychotic medications AE, adverse event; BACS, Brief Assessment of Cognition in Schizophrenia; BMI, body mass index; CGI-S, Clinical Global Impression–Severity scale; DSM, Diagnostic and Statistical Manual of Mental Disorders; DSM-IV, Diagnostic and Statistical Manual of Mental Disorders, Fourth Edition; DSM-IV-TR, Diagnostic and Statistical Manual of Mental Disorders, Fourth Edition, Text Revision; EudraCT, European Union Drug Regulating Authorities Clinical Trials database; FGA, first-generation antipsychotic; GAF, Global Assessment of Functioning; GLP-1RA, glucagon-like peptide-1 receptor agonist; ICD, International Classification of Diseases; ICD-10, International Classification of Diseases, 10th Revision; ITT, intention-to-treat; mITT, modified intention-to-treat; MCS, Mental Component Summary; MMRM, mixed-effects model for repeated measures; NCT, ClinicalTrials.gov identifier; PANSS, Positive and Negative Syndrome Scale; PANSS-6, six-item Positive and Negative Syndrome Scale; PCS, Physical Component Summary; PP, per-protocol; QoL, quality of life; RCT, randomized controlled trial; REDCap, Research Electronic Data Capture; REY, Rey Auditory Verbal Learning Test; SC, subcutaneous; SD, standard deviation; SGA, second-generation antipsychotic; SNAPSI, Simplified Negative and Positive Symptoms Interview; SQLS, Schizophrenia Quality of Life Scale; TAO, trial of exenatide in antipsychotic-treated obese patients. Values are presented as mean (SD) unless otherwise specified. Baseline BMI and clinical symptom scores are reported by treatment arm where available. “NOT REPORTED” indicates that the information was not available in the publication. For trials with multiple publications from the same parent trial, each publication is presented separately according to its reported role (primary or linked secondary analysis).

Study ID	Parent trial/trial grouping	Parent trial (registration)	Role	Year	Country	Setting	Design	Randomization ratio	Blinding	N randomized (GLP-1RA: comparator)	N analyzed (+ analysis)	Attrition (per arm)	Diagnosis	Diagnosis ascertainment (DSM/ICD + instrument)	Antipsychotic eligibility (agents)	Antipsychotic duration required at baseline	Mean age, y (SD)	% female	Baseline BMI, kg/m² (SD) per arm	Baseline PANSS or equivalent (SD) per arm	GLP-1RA agent	Dose & titration	Comparator	Treatment duration	Follow-up duration	Funding source
Ganeshalingam [1]	HISTORI trial	NCT05193578	Primary	2025	Denmark (Region of Southern Denmark and Region of Zealand)	Community-based outpatient mental health services; home-based blood sampling	Investigator-initiated, double-blind, parallel-group, superiority, multicentre RCT	0.042361	Double-blind; drug/placebo visually identical	77:77 (total 154)	ITT; completers 141	13/154 (semaglutide 3; placebo 10)	Schizophrenia, schizotypal disorder, schizoaffective disorder (ICD-10 F20/F21/F25)	Clinical ICD-10 diagnosis; PANSS-6 rated via SNAPSI interview	Second-generation antipsychotics	Stable SGA; >24 months in 91–93%	Overall 38.3 (10.7)	Overall 56.5%	40.66 / 40.16	PANSS-6 total: 20.05 / 21.71	Semaglutide	Up to 1.0 mg/week, titrated over 8 weeks	Matching placebo	30 weeks	30 weeks	Novo Nordisk Foundation; Steno Diabetes Center; other grants; Novo Nordisk supplied drug/placebo
Siskind [2]	COaST trial	ACTRN12621001539820	Primary	2025	Australia (Queensland)	Community/outpatient mental health and addiction services	Phase 2 multicentre participant- and investigator-blinded placebo-controlled RCT	0.042361	Participant and investigator masked	15:16 (total 31)	ITT; MMRM	1 semaglutide; 3 placebo	Schizophrenia (90%) or schizoaffective disorder (10%)	DSM-IV via Diagnostic Interview for Psychosis	Clozapine only	Clozapine ≥18 weeks	Overall 38.9	Overall 32%	36.51 / 36.89	PANSS total: 62.67 / 60.06	Semaglutide	Titrated to 2.0 mg/week	Placebo	36 weeks	36 weeks	NHMRC; Queensland Advancing Clinical Research Fellowship
Ishøy [3]	TAO trial	NCT01794429 / EudraCT 2012-005404-17	Primary (psychiatric/cognitive/QoL)	2017	Denmark	Psychiatric clinics	Investigator-initiated double-blind placebo-controlled RCT	0.042361	Double-blind	23:22 (total 45)	40 completers	Overall 11%	Schizophrenia-spectrum ICD-10 F20.x/F25.x	ICD-10 codes; no structured instrument reported	FGAs and SGAs	Stable antipsychotic ≥3 months	Completers: 37.4 / 34.4	Not reported	39.5 / 38.6	PANSS total: 63.7 / 65.9	Exenatide	2 mg weekly	Placebo	12–16 weeks	End of trial	University of Copenhagen; Lundbeck Foundation
Ishøy [4]	TAO trial (same cohort as Ishøy [3])	NCT01794429 / EudraCT 2012-005404-17	Linked secondary (metabolic/weight)	2017	Denmark	Psychiatric clinics	Same TAO RCT	0.042361	Double-blind	23:22 (total 45)	40 completers	Overall 11%	Same as TAO trial	Same as TAO trial	FGAs and SGAs	Stable antipsychotic ≥3 months	Randomized: 37.1 / 34.5	Not reported	39.2 / 38.4	See Ishøy [3]	Exenatide	2 mg weekly	Placebo	12–16 weeks	End of trial	As TAO
Larsen [5]	Larsen liraglutide trial	NCT01845259	Primary	2017	Denmark	Two psychiatric centres	Randomized double-blind placebo-controlled RCT	Approximately 1:1	Double-blind	52:51 (total 103)	mITT 47/50; safety ITT 52/51	Liraglutide 6; placebo 1	Schizophrenia-spectrum disorder	ICD-10 or DSM-IV-TR	Clozapine or olanzapine	Stable ≥6 months	Overall 42.5	Overall 41.7%	33.7 / 33.9	CGI-S: 3.6 / 3.8	Liraglutide	0.6→1.8 mg/day	Placebo	16 weeks	16 weeks	Novo Nordisk unrestricted grant; Lundbeck Foundation
Svensson [6]	Larsen liraglutide trial (same cohort as Larsen [5])	NCT01845259	Linked secondary (68-week follow-up)	2019	Denmark	Two psychiatric centres	One-year naturalistic follow-up of Larsen RCT	As parent trial	Blinding extended to week 68	103 (52:51)	mITT week 68	8.3% dropout	Same as Larsen trial	Same as Larsen trial	Clozapine or olanzapine	≥6 months	Per parent trial	Per parent trial	Per parent trial	CGI-S / SQLS / GAF	Liraglutide	0.6→1.8 mg/day	Placebo weeks 0–16; usual care thereafter	16 weeks + follow-up	Week 68	As parent trial
Ganeshalingam [7]	HISTORI trial (same cohort as Ganeshalingam [1])	NCT05193578	Linked secondary (metabolic)	2026	Denmark	Community-based outpatient services; home-based sampling	Secondary prespecified metabolic analysis of HISTORI RCT	0.042361	Double-blind	77:77 (total 154)	ITT; insulin data available for n=131	13/154	Schizophrenia, schizotypal disorder, schizoaffective disorder	Clinical ICD-10 diagnosis	Second-generation antipsychotics	Stable SGA; >24 months in 91–93%	Overall 38.3 (10.7)	0.565	40.66 / 40.16	See parent trial	Semaglutide	Up to 1.0 mg/week over 8 weeks	Matching placebo	30 weeks	30 weeks	As HISTORI
Uhrenholt [8]	HISTORI trial (same cohort as Ganeshalingam [1])	NCT05193578	Linked secondary (mediation analysis)	2026	Denmark	Community-based outpatient services	Secondary causal mediation analysis of HISTORI RCT	0.042361	Double-blind	77:77 (total 154)	ITT (n=154)	As HISTORI	Schizophrenia, schizotypal disorder, schizoaffective disorder	Clinical ICD-10; PANSS-6 via SNAPSI	Second-generation antipsychotics	Stable SGA; >24 months in 91–93%	Overall 38.3 (10.7)	0.565	40.66 / 40.16	PANSS-6 total: 20.05 / 21.71	Semaglutide	Up to 1.0 mg/week over 8 weeks	Matching placebo	30 weeks	30 weeks	As HISTORI

Characteristics of Included Studies

The four RCTs included 333 participants: COaST (n=31), TAO (n=45), the Larsen trial (n=103), and HISTORI (n=154) [1-6]. All trials were double-blind, placebo-controlled studies evaluating a GLP-1RA added to ongoing antipsychotic treatment in adults with schizophrenia-spectrum disorders and overweight or obesity [1-6]. Two trials required prediabetes at baseline [1,5]. The evaluated agents were exenatide, liraglutide, and semaglutide, with treatment durations ranging from 12 to 36 weeks and an additional 68-week follow-up period in the Larsen trial [1-6]. Antipsychotic eligibility varied from clozapine-specific treatment to any antipsychotic use [1-6]. Psychiatric symptoms, cognition, and quality-of-life outcomes were secondary or exploratory outcomes in all trials, whereas metabolic outcomes represented the primary endpoints [1-6].

Psychiatric symptoms were assessed using the Positive and Negative Syndrome Scale (PANSS) [11], Clinical Global Impression-Severity (CGI-S) scale [12], or six-item PANSS (PANSS-6) [13]. Cognition was assessed in two trials using the Brief Assessment of Cognition in Schizophrenia (BACS) [14]. Quality of life and functioning were measured using the 36-item Short-Form Health Survey (SF-36/SF-36v2) [15], Personal and Social Performance scale [16], and Schizophrenia Quality of Life Scale [17].

Risk-of-Bias Assessment

Across 11 outcome-domain assessments, no assessment was judged to have a high risk of bias. One assessment was rated as low risk, while the remainder raised some concerns [1-6]. Concerns were mainly related to attrition, analysis populations, and selective reporting. The TAO trial used completer/per-protocol analyses for some outcomes [3,4], the Larsen and HISTORI trials had differential attrition between groups [1,5,6], and some exploratory quality-of-life analyses involved multiple uncorrected comparisons [3]. Outcome measurement was considered low risk across trials because assessments were based on validated instruments administered under blinded conditions (Table 2) [1-6].

**Table 2:** Risk-of-bias assessment of included randomized controlled trials by outcome domain D1, bias arising from the randomization process; D2, bias due to deviations from intended interventions; D3, bias due to missing outcome data; D4, bias in measurement of the outcome; D5, bias in selection of the reported result; RCT, randomized controlled trial; RoB 2, Cochrane Risk of Bias 2 tool; GLP-1RA, glucagon-like peptide-1 receptor agonist Risk-of-bias judgements are reported as “Low”, “Some concerns”, or “Not assessed”. “Not assessed” indicates that an outcome-specific risk-of-bias judgement was not available from the provided assessment data and was not inferred. For studies with multiple publications from the same parent trial, each publication was linked to the corresponding study reference number and assessed according to the reported outcome domain.

Study ID	Outcome domain	D1	D2	D3	D4	D5	Overall
Ganeshalingam [1]	Psychiatric	Some concerns	Low	Some concerns	Low	Low	Some concerns
Ganeshalingam [1]	Quality of life and functioning	Some concerns	Low	Some concerns	Low	Low	Some concerns
Siskind [2]	Psychiatric	Low	Low	Low	Low	Low	Low
Siskind [2]	Cognition	Low	Low	Low	Low	Some concerns	Some concerns
Ishøy [3]	Psychiatric	Low	Some concerns	Some concerns	Low	Some concerns	Some concerns
Ishøy [3]	Cognition	Low	Some concerns	Some concerns	Low	Low	Some concerns
Ishøy [3]	Quality of life and functioning	Low	Some concerns	Some concerns	Low	Some concerns	Some concerns
Ishøy [4]	Metabolic/weight	Not assessed	Not assessed	Not assessed	Not assessed	Not assessed	Not assessed
Larsen [5]	Psychiatric	Low	Low	Some concerns	Low	Low	Some concerns
Larsen [5]	Quality of life and functioning	Low	Low	Some concerns	Low	Low	Some concerns
Svensson [6]	Psychiatric	Low	Some concerns	Some concerns	Low	Low	Some concerns
Svensson [6]	Quality of life and functioning	Low	Some concerns	Some concerns	Low	Low	Some concerns
Ganeshalingam [7]	Metabolic/insulin sensitivity	Not assessed	Not assessed	Not assessed	Not assessed	Not assessed	Not assessed
Uhrenholt [8]	Quality of life and psychopathology (mediation analysis)	Not assessed	Not assessed	Not assessed	Not assessed	Not assessed	Not assessed

Narrative Synthesis

Meta-analysis was not performed because no outcome had at least three trials reporting comparable measures with extractable variance data. Findings were therefore synthesized narratively by outcome domain using the direction and statistical significance of effects, supported by reported estimates and 95% confidence intervals (CIs).

Psychiatric Symptom Severity

All trials (333 randomized participants) found no statistically significant effect of GLP-1RAs on psychiatric symptom severity [1-6]. In COaST, semaglutide did not affect the PANSS total score or subscales (between-group difference 0.20; P=0.95) [2]. In TAO, exenatide showed no treatment-by-time effect on the PANSS total score (P=0.86) [3,4]. In the Larsen trial, liraglutide did not improve CGI-S scores at 16 weeks (−0.1; 95% CI −0.3 to 0.2; P=0.49), and the 68-week follow-up remained statistically non-significant (−0.5; 95% CI −0.9 to 0.01; P=0.05) [5,6]. HISTORI reported no significant effect of semaglutide on PANSS-6 scores [1]. Overall, there was no consistent evidence that GLP-1RAs improved or worsened psychiatric symptoms, with low-to-moderate certainty of evidence.

Cognition

Two trials assessed cognition (76 randomized participants) [2,3]. Neither demonstrated a pro-cognitive effect. In TAO, the BACS composite score, which was the primary cognitive outcome, showed no treatment-by-time interaction (P=0.77), with no significant effects across subtests [3]. COaST also reported no significant cognitive benefit based on BACS and Trail Making Test assessments [2]. Certainty of evidence was low because of the small number of trials and incomplete numerical reporting in one study.

Quality of Life and Functioning

Three trials assessed quality of life or functioning (302 randomized participants) [1,3,5,6]. HISTORI reported improved physical quality of life with semaglutide, measured using the SF-36v2 Physical Component Summary (+3.75 points; 95% CI 1.52 to 5.98; P=0.001), while mental quality of life remained unchanged [1]. A mediation analysis suggested that this improvement was largely explained by weight loss rather than a direct psychotropic effect [8].

In TAO, one SF-36 subscale showed improvement with exenatide; however, this finding was based on exploratory uncorrected testing and was not supported by other quality-of-life or functioning measures [3,4]. The Larsen trial found no improvement in the Schizophrenia Quality of Life Scale or Global Assessment of Functioning at either 16 weeks or 68 weeks [5,6]. Overall, evidence for quality-of-life benefit was limited to physical health outcomes and was considered low certainty.

Across four RCTs, GLP-1RAs added to antipsychotic treatment showed no evidence of benefit or harm regarding psychiatric symptoms or cognition. The only observed quality-of-life benefit was improvement in physical health, which appeared to be mediated by weight loss rather than a direct psychiatric effect [1-8].

Limitations

Several limitations should be considered. First, neuropsychiatric outcomes were secondary or exploratory endpoints in all included trials, and none were specifically powered to detect changes in psychiatric symptoms, cognition, or quality of life. Second, the evidence base was small and heterogeneous, with variation in GLP-1RA agents, doses, treatment durations, antipsychotic regimens, and outcome measures, preventing quantitative synthesis. Third, incomplete reporting of variance data limited the ability to compare effects across studies. Fourth, some trials had methodological limitations, including small sample sizes, attrition, and concerns regarding selective reporting. Finally, most outcome assessments raised some concerns regarding risk of bias, highlighting the need for larger and more definitive studies.

Implications

GLP-1RAs appear to have a role as metabolic adjuncts for individuals with schizophrenia-spectrum disorders receiving antipsychotic treatment, particularly for reducing weight-related health risks. Current evidence does not support their use as treatments for psychiatric symptoms or cognitive impairment; however, it provides reassurance that they do not appear to worsen mental state. Future trials should prioritize neuropsychiatric outcomes, use longer follow-up periods, apply harmonized assessment tools, and report complete statistical data to enable more robust evidence synthesis.

## Conclusions

In adults with schizophrenia-spectrum disorders receiving antipsychotic treatment, GLP-1 receptor agonists should be considered primarily as metabolic interventions rather than treatments for psychiatric or cognitive symptoms. Current evidence does not indicate meaningful effects on mental-state outcomes, while potential benefits appear to relate mainly to physical health. Further research using rigorous designs, longer follow-up periods, and comprehensive assessment of neuropsychiatric outcomes is needed to clarify their broader clinical role.

## Appendices

**Table 3:** Complete electronic search strategies No restrictions on publication date; human studies only where database filters were available; English-language publications Randomized controlled trials were selected during study screening rather than by applying database filters.

Database	Search date	Complete search strategy
MEDLINE (via PubMed)	46223	("Glucagon-Like Peptide 1 Receptor Agonists"[Mesh] OR "glucagon-like peptide-1 receptor agonist*" OR GLP-1RA* OR GLP-1 OR semaglutide OR liraglutide OR exenatide OR dulaglutide OR lixisenatide OR tirzepatide) AND ("Schizophrenia"[Mesh] OR schizophrenia OR schizophren* OR schizoaffective OR schizotypal OR "schizophrenia spectrum disorder*") AND ("Antipsychotic Agents"[Mesh] OR antipsychotic* OR neuroleptic* OR clozapine OR olanzapine OR risperidone OR quetiapine OR aripiprazole OR amisulpride OR ziprasidone OR paliperidone)
Cochrane CENTRAL	46223	("glucagon-like peptide-1 receptor agonist*" OR GLP-1RA* OR GLP-1 OR semaglutide OR liraglutide OR exenatide OR dulaglutide OR lixisenatide OR tirzepatide) AND (schizophrenia OR schizophren* OR schizoaffective OR schizotypal OR "schizophrenia spectrum") AND (antipsychotic* OR neuroleptic* OR clozapine OR olanzapine)
Scopus	46223	TITLE-ABS-KEY(("glucagon-like peptide-1 receptor agonist*" OR GLP-1RA* OR GLP-1 OR semaglutide OR liraglutide OR exenatide OR dulaglutide OR lixisenatide OR tirzepatide) AND (schizophrenia OR schizophren* OR schizoaffective OR schizotypal OR "schizophrenia spectrum") AND (antipsychotic* OR neuroleptic* OR clozapine OR olanzapine))
Web of Science Core Collection	46223	TS=(("glucagon-like peptide-1 receptor agonist*" OR GLP-1RA* OR GLP-1 OR semaglutide OR liraglutide OR exenatide OR dulaglutide OR lixisenatide OR tirzepatide) AND (schizophrenia OR schizophren* OR schizoaffective OR schizotypal OR "schizophrenia spectrum") AND (antipsychotic* OR neuroleptic* OR clozapine OR olanzapine))

## References

[1] Ganeshalingam AA, Uhrenholt N, Arnfred S (2025). Semaglutide treatment of antipsychotic-treated patients with schizophrenia, prediabetes, and obesity. The HISTORI randomized clinical trial. JAMA Psychiatry.

[2] Siskind D, Baker A, Arnautovska U (2025). Efficacy and safety of semaglutide versus placebo for people with schizophrenia on clozapine with obesity (COaST): a phase 2, multi-centre, participant and investigator- blinded, randomised controlled trial in Australia. Lancet Psychiatry.

[3] Ishøy PL, Fagerlund B, Broberg BV, Bak N, Knop FK, Glenthøj BY, Ebdrup BH (2017). No cognitive-enhancing effect of GLP-1 receptor agonism in antipsychotic-treated, obese patients with schizophrenia. Acta Psychiatr Scand.

[4] Ishøy PL, Knop FK, Broberg BV (2017). Effect of GLP-1 receptor agonist treatment on body weight in obese antipsychotic-treated patients with schizophrenia: a randomized, placebo-controlled trial. Diabetes Obes Metab.

[5] Larsen JR, Vedtofte L, Jakobsen MS (2017). Effect of liraglutide treatment on prediabetes and overweight or obesity in clozapine- or olanzapine-treated patients with schizophrenia spectrum disorder: a randomized clinical trial. JAMA Psychiatry.

[6] Svensson CK, Larsen JR, Vedtofte L (2019). One-year follow-up on liraglutide treatment for prediabetes and overweight/obesity in clozapine- or olanzapine-treated patients. Acta Psychiatr Scand.

[7] Page MJ, McKenzie JE, Bossuyt PM (2021). The PRISMA 2020 statement: an updated guideline for reporting systematic reviews. Rev Esp Cardiol (Engl Ed).

[8] Schardt C, Adams MB, Owens T, Keitz S, Fontelo P (2007). Utilization of the PICO framework to improve searching PubMed for clinical questions. BMC Med Inform Decis Mak.

[9] Sterne JA, Savović J, Page MJ (2019). RoB 2: a revised tool for assessing risk of bias in randomised trials. BMJ.

[10] Guyatt GH, Oxman AD, Schünemann HJ, Tugwell P, Knottnerus A (2011). GRADE guidelines: a new series of articles in the Journal of Clinical Epidemiology. J Clin Epidemiol.

[11] Opler MGA, Yavorsky C, Daniel DG (2017). Positive and Negative Syndrome Scale (PANSS) training: challenges, solutions, and future directions. Innov Clin Neurosci.

[12] Busner J, Targum SD (2007). The clinical global impressions scale: applying a research tool in clinical practice. Psychiatry (Edgmont).

[13] Kølbæk P, Dines D, Hansen J, Opler M, Correll CU, Mors O, Østergaard SD (2021). Standardized training in the rating of the six-item Positive And Negative Syndrome Scale (PANSS-6). Schizophr Res.

[14] Keefe RS, Goldberg TE, Harvey PD, Gold JM, Poe MP, Coughenour L (2004). The Brief Assessment of Cognition in Schizophrenia: reliability, sensitivity, and comparison with a standard neurocognitive battery. Schizophr Res.

[15] Jenkinson C, Coulter A, Wright L (1993). Short form 36 (SF36) health survey questionnaire: normative data for adults of working age. BMJ.

[16] Juckel G, Schaub D, Fuchs N (2008). Validation of the Personal and Social Performance (PSP) Scale in a German sample of acutely ill patients with schizophrenia. Schizophr Res.

[17] Wilkinson G, Hesdon B, Wild D (2000). Self-report quality of life measure for people with schizophrenia: the SQLS. Br J Psychiatry.

